# Reassortment and adaptive mutations of an emerging avian influenza virus H7N4 subtype in China

**DOI:** 10.1371/journal.pone.0227597

**Published:** 2020-01-17

**Authors:** Bingqian Qu, Xue Li, Carol J. Cardona, Zheng Xing

**Affiliations:** 1 Medical School and the Jiangsu Provincial Key Laboratory of Molecular Medicine, Nanjing University, Nanjing, China; 2 Department of Veterinary Biomedical Sciences, College of Veterinary Medicine, University of Minnesota at Twin Cities, Saint Paul, MN, United States of America; Icahn School of Medicine at Mount Sinai, UNITED STATES

## Abstract

Human infections with avian influenza viruses including H5, H7 and H9 hemagglutinin subtypes occur at a low rate. Among human infections with H7 viruses, regional outbreaks with H7N2, H7N3, H7N7 and H7N9 have been documented. Early in 2018, a human infection with a novel H7N4 avian influenza virus was reported in Jiangsu, China. This study is aimed at understanding the probable origin and molecular features of this emerging H7N4 virus. Genomic segments encoding hemagglutinin (HA) and neuraminidase (NA) of H7Nx and HxN4 viruses were compared with this H7N4 strain by alignment and phylogenetic tree analysis. Phylogenetic analysis indicated that the human H7N4 virus probably originated from multiple reassortments of avian H7N7 and H8N4 viruses for its HA and NA, respectively, and likely a regional uncharacterized virus for its internal segments. Our data excluded that circulating avian H9N2 viruses were the origin of the H7N4 internal segments, unlike the human H5N1 and H7N9 viruses that both had H9N2 backbones. This index case provided a unique opportunity to examine viral mutations by directly comparing the human isolate with its closest viral relatives isolated from avian species from the patient’s farm, which may suggest critical mutations required for viral adaptation in humans. Whole-genome scanning was performed and the sequences of the human and related avian H7N4 isolates were compared. Mutations in PB2 (E627K), PB2 (K683T), PB1-F2 (N47S), HA (N283D), HA(K321E), NA(A137V), NA(K296R) and M2 (C19Y) were identified in the human isolate while no mutations were found in PB1, NP, NS1, and NS2 of the human H7N4 compared to the avian H7N4 viruses. Our data in this report provide further evidence for the genesis of this novel H7N4 virus with a multi-reassortment model and show molecular changes that might be responsible for the transmission of this virus from chickens or ducks to and subsequent replication in humans.

## Introduction

Influenza A virus is classified into 18 hemagglutinin (HA) and 11 neuraminidase (NA) subtypes [1]. Human infections with avian influenza viruses (AIV) do occur, albeit at a very low rate. Some avian viruses, including H5 or H7 subtypes, have crossed species barriers with acquired ability from mutations and caused sporadic infections or regional and even intercontinental outbreaks in humans with high pathogenicity and fatality rates [2]. Among human infections with H7 viruses, geographical distribution showed that infections with H7N2 (New York) and H7N3 (Canada, Mexico) were the only documented cases in Northern America, whereas H7N7 outbreaks occurred in England, the Netherlands, and Italy [3]. Human infections with a novel H7N9 virus was first reported in China, 2013, and has since become a severe public health threat with high mortality rates. So far the H7N9 virus had caused more than a thousand infections and drawn global concern due to its pandemic potential [4]. To date, other H7 subtype viruses have not been detected in humans.

Little was known about the H7N4 virus until an outbreak of highly pathogenic avian influenza occurred in chickens in Australia in 1997 [5]. In early 2018, a severe H7N4 infection was confirmed in a 68-year-old woman in Jiangsu, China, which was the first and only human case infected with an H7N4 as shown in the previous studies [6, 7]. The patient was cured with oseltamivir, glucocorticoid, and antibiotic treatment after hospitalization including a week in an intensive care unit [6, 7]. After oseltamivir treatment, the virus could no longer be isolated from throat swabs but full genome sequences of the virus were obtained by next-generation sequencing with samples from the patient. In this report, we expand on what was learned from the previous reports [6, 7] and performed additional analyses on the internal gene segments of the human H7N4 virus, which suggested a model for multi-reassortment of the new virus based on phylogenetic analyses and current knowledge of the wild bird migratory routes. Unlike the highly pathogenic H5N1 and emerging H7N9 AIV, the novel H7N4 virus appeared not to be derived or obtain the internal genomic segments from the endemically circulating H9N2 and H7N9 viruses. In addition, we compared the genomic sequences of the human virus and the viruses of the H7N4 subtype isolated from avian species concurrently circulating in the same farm, which provided critical information about potential adaptive mutations potentially required for the novel H7N4 capable of being transmitted and replicative in humans. We conclude that the human-origin H7N4 virus probably originated from a reassortment of H7N7, H8N4, and likely H5N1 viruses without contribution from circulating H9N2 pools. By comparing the virus with the avian viruses isolated on the same farm, we identified seventeen probable human-adaptive mutations, most of which were uncharacterized except the E627K and K683T mutations in PB2.

## Materials and methods

### Genomic and protein sequences

Entire genome and protein sequences of A/Jiangsu/1/2018 (H7N4) (Isolate ID: EPI_ISL_291111) and close-related strains, that were isolated at the same farm including A/chicken/Jiangsu/1/2018 (H7N4) (ID: EPI_ISL_291131), A/chicken/Jiangsu/103/2018 (H7N4) (ID: EPI_ISL_293286), A/duck/Jiangsu/4/2018 (H7N4) (ID: EPI_ISL_293287), A/duck/Jiangsu/8/2018 (H7N4) (ID: EPI_ISL_293288), A/duck/Jiangsu/12/2018 (H7N4) (ID: EPI_ISL_293289), A/duck/Jiangsu/13/2018 (H7N4) (ID: EPI_ISL_293290), and A/duck/Jiangsu/16/2018 (H7N4) (ID: EPI_ISL_293291), were downloaded from the GISAID database [8]. As reference strains, A/Anhui/1/2013 (H7N9) (ID: EPI_ISL_138739), A/Shanghai/1/2013 (H7N9) (ID: EPI_ISL_138737), A/Shanghai/1/2006 (H5N1) (ID: EPI_ISL_64227), and A/chicken/Jiangsu/YB7/2015 (H5N1) (ID: EPI_ISL_266437) were included for analyses.

### Phylogenetic analysis of HA and NA genes

Hemagglutinin sequences representing H7N1~H7N9 strains were collected (GISAID), aligned, and constructed into phylogenetic trees using the neighbor-joining algorithm with 1000 bootstrap replications (MEGA6 software) [9]. Similarly, the NA segments of the HxN4 isolates, including strains of H1N4~H12N4, H14N4 and H15N4, but not H13N4, were analyzed. The H13N4 NA sequence was not available in two databases, GISAID and Fludb, in which only an incomplete HA sequence was available from a sole H13N4 virus. In both analyses, a non-H7 and non-N4 viral strain, A/chicken/Jiangsu/YB7/2015 (H5N1) (ID: EPI_ISL_266437), was selected as an outgroup control.

### Phylogenetic analysis of six internal genes

**Fig 1:**
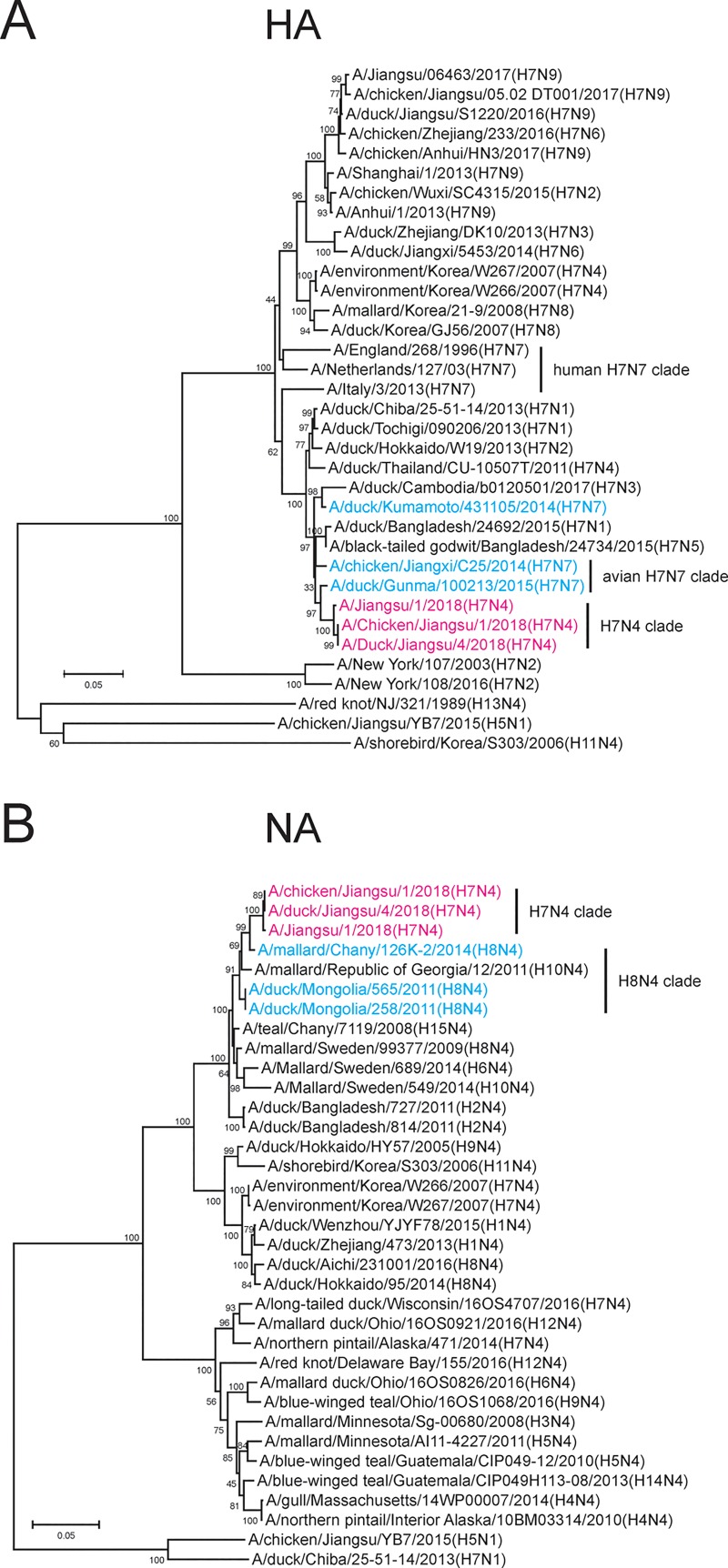
Phylogenetic tree analysis of the origins of HA and NA segments of the H7N4 human isolate in China. **A**) 35 HA segments of H7 isolates were analyzed using MEGA6 software. Magenta: new H7N4 viruses including A/Jiangsu/1/2018 (JS2018); Cyan: related avian H7N7 viruses; Black: H7N1, H7N2, H7N3, H7N5, H7N6, H7N7 (human), H7N8 and H7N9 viruses. A/red not/NJ/321/1989 (H13N4), A/shorebird/Korea/S303/2006 (H11N4) and A/chicken/Jiangsu/YB7/2015 (H5N1) isolated in Jiangsu were used as outgroup control strains. Scale bar indicates the amount of substitutions in the nucleotides. **B**) 35 NA segments of N4 isolates were analyzed using MEGA6. Magenta: H7N4 viruses including JS2018; Cyan: related avian H8N4 viruses; Black: H1N4, H2N4, H3N4, H4N4, H5N4, H6N4, H8N4, H9N4, H10N4, H11N4, H12N4, H14N4, and H15N4 viruses. Outgroup controls: A/chicken/Jiangsu/YB7/2015 (H5N1) and A/duck/Chiba/25-51-14/2013 (H7N1). Scale bar: the amount of nucleotide substitutions.

Six internal genomic fragments of the viruses, including A/Jiangsu/1/2018 (H7N4), A/chicken/Jiangsu/1/2018 (H7N4), A/duck/Jiangsu/4/2018 (H7N4), avian H7N7, and H8N4 viruses, identified in Fig 1, representative avian H9N2 viruses (BJ/94-like, G1-like, F/98-like, and local ones in Jiangsu province isolated from 2000 to 2015), and A/chicken/Jiangsu/YB7/2015, were downloaded from the NCBI database. The sequences of their PB2, PB1, PA, NP, M, and NS segments were aligned by Clustal W, embedded in the MEGA6 software, and subjected to phylogenetic analysis as described above.

### Analysis of human adaptive mutations in the index case

To analyze adaptive mutations of this novel H7N4 virus in humans, we performed whole-genome scanning of the human H7N4 virus along with all other avian H7N4 viruses isolated from the same farm. Mutations were thereafter identified and further compared with other selected viruses to determine possible mutations that might be relevant to the human adaptation of this H7N4 virus.

## Results

### Human-origin H7N4 virus is a reassortant of avian H7N7 and H8N4 viruses

Sequences of the viruses, A/Jiangsu/1/2018 (JS2018) and the strains from chickens and ducks raised by the patient, A/chicken/Jiangsu/1/2018 (Ck2018), and A/duck/Jiangsu/4/2018 (Dk2018), were analyzed (MEGA6 software). A phylogenetic tree analysis showed that the HA of a novel H7N4 cluster was distant from recent human and avian H7N9 sequences as well as other avian H7N4 isolates but was highly similar (>98%) to avian H7N7 viruses from Japan, suggesting an origin from a geographically distant location (Fig 1A). On the other hand, the NA of JS2018, Ck2018, and Dk2018 had low similarity to a Korean H7N4 isolate, A/environment/Korea/W266/2007, but was similar to avian H8N4 isolates from Chany and Mongolia (>97%) (Fig 1B). Both the HA and NA genes belonged to the Eurasian lineages of AIV.

### Six internal genomic segments of the human H7N4 virus were derived from H7N7, H8N4 and H5N1 but not circulating H9N2 viruses

**Fig 2:**
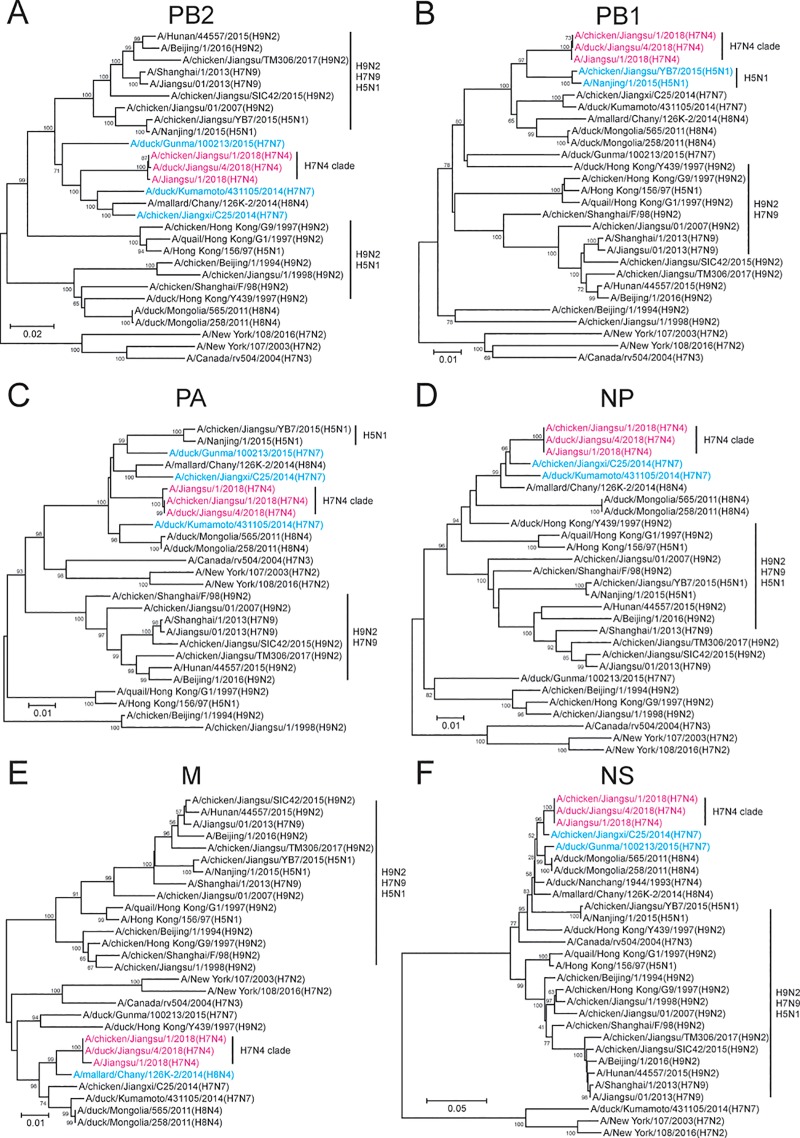
Phylogeny of the origins of PB2, PB1, PA, NP, M and NS segments of the novel H7N4 virus from H7N7, H8N4, and H5N1 viruses. The internal segments of the viruses were aligned and subjected to the construction of phylogenetic trees. The viruses included JS2018, A/chicken/Jiangsu/1/2018, A/duck/Jiangsu/4/2018, H7N7, and H8N4 strains identified in Fig 1, and representative avian H9N2 strains as well as human H5N1, H7N9, H7N2 and H9N2 viruses, collected in mainland China between 1994 and 2017. **A)** PB2; **B)** PB1; **C)** PA; **D)** NP; **E)** M; and **F)** NS. H7N4 viruses (magenta) and related H7N7, H8N4 and H5N1 viruses (cyan) were highlighted. For each segment, the scale bar indicates the number of nucleotide substitutions. The human H7N3 virus (A/Canada/rv504/2004 (H7N3)), which was never identified in Europe and Asia, was used as outgroup control.

We next investigated the origin of the internal genomic segments in the human H7N4 virus (JS2018). We compared each of the six gene segments of the human and domestic avian H7N4 viruses from the patient’s farm with published H7N4, H7N7, H8N4 sequences, and multiple avian H9N2 viral sequences from a variety of regions collected over the past twenty years. Circulating H9N2 viruses have been a pool of influenza genes contributing internal segments to both highly pathogenic H5N1 and emerging H7N9 viruses [10, 11]. However, the internal gene segments of the human and avian H7N4 viruses had little similarity to those of circulating H9N2 viruses (Fig 2). Interestingly, we found that most of the internal gene segments (PB2, PA, NP, M, and NS) of the human and avian H7N4 viruses were similar to those of local (A/chicken/Jiangxi/C25/2014) or Japanese (A/duck/Kumamoto/431105/2014) H7N7 and Russian (A/mallard/Chany/126K-2/2014) H8N4 viruses (Fig 2A and 2C–2F). The PB1 segment of the novel H7N4 viruses was closely related to that of an H5N1 virus (A/chicken/Jiangsu/YB7/2015) in Jiangsu (Fig 2B). In summary, our analysis indicated that the internal gene segments of these H7N4 viruses, unlike those of H5N1 and H7N9, were likely derived from uncharacterized local avian viral strains which were not circulating H9N2 viruses.

**Fig 3:**
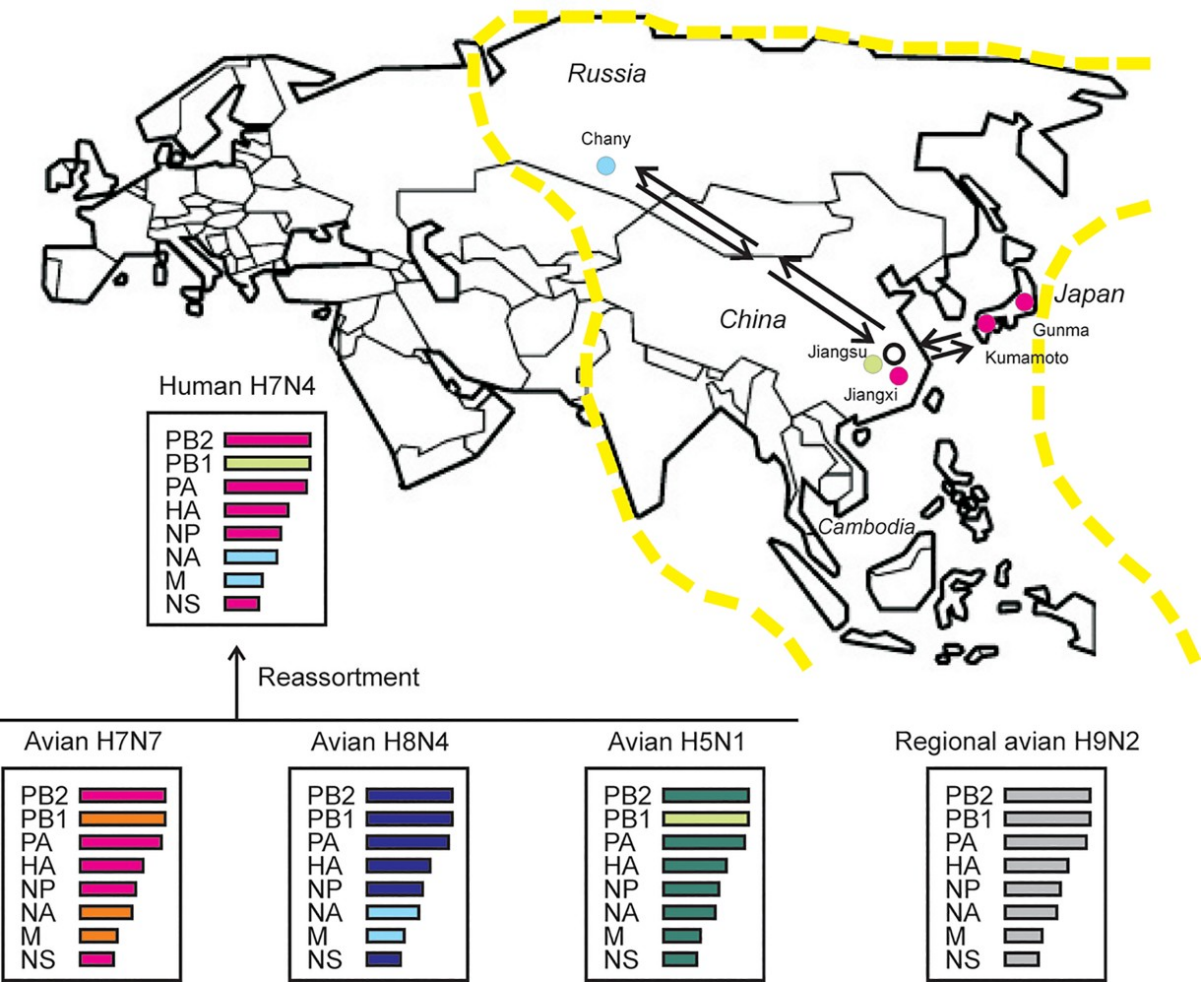
Probable multi-reassortment of the novel human H7N4 virus. For JS2018, the PB2, PA, HA, NP, and NS segments (magenta) were avian H7N7-derived; The NA and M segments (cyan) were donated by an avian H8N4 virus; PB1 (green) was likely obtained from an avian H5N1 virus. Remarkably, none of these internal segments was acquired from any known regional H9N2 viruses (grey). Possible migratory route of birds carrying H7N7 or H8N4 viruses was shown in arrows in the map. Magenta dot: Gunma (Japan), Kumamoto (Japan), Jiangxi (China). Cyan dot: Chany (Russia). Green dot: Changzhou, Jiangsu (China). Yellow dashline: the East Asia-Australia migratory flyway.

Jiangsu is located at the route of the East Asian-Australian migratory flyway where avian influenza viral reassortments have been frequently detected [12]. Based on the evidence that neither the HA nor the NA of the H7N4 virus was from known H7 or N4 lineages, we considered that this was a novel reassortant, likely out of a multi-reassortment event. We hypothesized that the multi-reassortment took place when an H7N7 virus from a bird from East Asia and an H8N4 virus carried by a migratory bird from Siberia, contributed the HA and NA, respectively, infected a local bird in Jiangsu province, which was co-infected with a local uncharacterized viral strain then supplied the reassortant with its internal gene segments, including PB1, PB2, PA, HA, NP, and NS. However, at this stage, we were unable to provide an exact temporal order how the reassortment event occurred (Fig 3).

### Molecular features of the human H7N4 virus

We wondered how an avian-origin H7N4 virus adapted to a human host. First, we compared the molecular characteristics of JS2018, and the two related chicken and five duck isolates with those of A/Anhui/1/2013 (H7N9), A/Shanghai/1/2013 (H7N9), and A/Shanghai/1/2006 (H5N1). In the HAs, all H7N4 and A/Shanghai/1/2013 viruses retained avian receptor binding preference and had the same Q226-S227-G228 H3 numbering motif in the receptor-binding site. JS2018 had a low pathogenic cleavage site of PELPKGR/GLF. A unique feature of JS2018 among the novel H7N4 isolates was its E627K in PB2, similar to the H7N9 virus, a mutation that is thought to increase virulence [13]. None of the novel H7N4 viruses would be resistant to amantadine as no S31N in M2 was observed. Strikingly, no H274Y or R292K in NA changes, which had been associated with oseltamivir resistance, were identified in any of the H7N4 isolates, and not in A/Anhui/1/2013 or partly in A/Shanghai/1/2013, which explained the patient’s positive response to oseltamivir treatment and full recovery. In fact according to the original report the position at NA residue 292 was present with a mixture of R (65%) and K (35%) [14], likely indicating an intermediate state during the viral evolution. The NA stalk deletion found in H5N1 and two H7N9 viruses was not observed in any of the H7N4 isolates. Additionally, all H7N4 isolates showed avian viral features without any deletions in NS1 and PB1-F2 genes (Table 1).

**Table 1:** Molecular features of the human and related domestic avian H7N4 isolates compared to human H7N9 and H5N1 strains.

Virus	Subtype	PB2			PB1		PB1-F2		PA			HA		NA			M1		M2	NS1	
		Virulence					del.					CS	RBS	Stalk	Resistance*		Virulence		Resistance§	del.	
		89	627	701	99	368		66	100	356	409			del.	274	292	30	218	31		42
JS2018	H7N4	V	**K**	D	H	I	90aa	N	V	K	S	PELPKGR/GLF	QSG	No	H	R	D	T	S	230aa	S
Ck2018	H7N4	V	E	D	H	I	90aa	N	V	K	S	PELPKGR/GLF	QSG	No	H	R	D	T	S	230aa	S
A/Ck/103/2018	H7N4	V	E	D	H	I	90aa	N	V	K	S	PELPKGR/GLF	QSG	No	H	R	D	T	S	230aa	S
Dk2018	H7N4	V	E	D	H	I	90aa	N	V	K	S	PELPKGR/GLF	QSG	No	H	R	D	T	S	230aa	S
A/Dk/8/2018	H7N4	V	E	D	H	I	90aa	N	V	K	S	PELPKGR/GLF	QSG	No	H	R	D	T	S	230aa	S
A/Dk/12/2018	H7N4	V	E	D	H	I	90aa	N	V	K	S	PELPKGR/GLF	QSG	No	H	R	D	T	S	230aa	S
A/Dk/13/2018	H7N4	V	E	D	H	I	90aa	N	V	K	S	PELPKGR/GLF	QSG	No	H	R	D	T	S	230aa	S
A/Dk/16/2018	H7N4	V	E	D	H	I	90aa	N	V	K	S	PELPKGR/GLF	QSG	No	H	R	D	T	S	230aa	S
A/Anhui/1/2013	H7N9	V	**K**	D	H	**V**	90aa	N	**A**	**R**	**N**	PE**I**PKGR/GLF	**L**SG	Yes	H	R	D	T	**N**	217aa	S
A/SH/1/2013	H7N9	V	**K**	D	H	I	90aa	N	**A**	**R**	**N**	PE**I**PKGR/GLF	QSG	Yes	H	**R/K**^#^	D	T	**N**	217aa	S
A/SH/1/2006	H5N1	V	E	D	H	I	90aa	N	V	K	S	PLRERRRKRGLF	QSG	Yes	H	R	D	T	S	230aa	S

JS: Jiangsu; Ck: chicken; Dk: duck; SH: shanghai; del.: deletion; aa: amino acids; CS: cleavage site; RBS: receptor binding site

*: oseltamivir

§: amantadine. Bold character: mutated residues.

#Yen HL, et al. [14].

### Multiple probable human adaptive mutations are identified in the human H7N4 virus

Transmission of the H7N4 virus to a human within the patient’s farm setting provided an opportunity to study critical adaptive mutations by comparing the various isolates from different species. We analyzed the whole genome of JS2018 for differences with the Ck2018 (from chickens) and Dk2018 (from ducks) isolated from the source farm, as well as other avian H7N4 isolates. The data were further compared with the sequences and mutations identified in human H7N9 and H5N1 isolates, which led to human infection in the same geographic region in China, in order to identify mutations in AIVs critical for human adaptation.

In the H7N4 viruses, we identified 17 mutations in seven viral proteins (5 in PB2, 1 in PB1-F2, 2 in PA, 5 in HA, 2 in NA, 1 in M1, and 1 in M2). No mutations were detected in PB1, NP, and surprisingly non in NS1, or NS2. Overall, JS2018 had the highest similarity to one of the chicken isolates, A/chicken/Jiangsu/103/2018, indicating potential transmission to the patient from chickens. Again, JS2018 was the only H7N4 isolate with the E627K mutation in PB2 which was also present in human H7N9 viruses. In addition, a PB2 K683T mutation occurred in JS2018 and Ck2018 but not in the duck H7N4 viruses. Of special note, four mutations in the JS2018 HA (H242Q, N283D, K321E, and I479M) were identified. We also noted that an N47S mutation in PB1-F2 was common to all human H7N4, H7N9 and H5N1 isolates, and a C19Y mutation in M2 occurred only in this human H7N4 isolate (Table 2). These unique changes in JS2018 may play a critical role potentially in transmission and adaptation of the novel H7N4 from avian species to humans.

**Table 2:** Whole-genome mutations in the human H7N4 isolate compared with related H7N4 avian isolates and human H7N9 and H5N1 isolates.

Virus	Subtype	PB2					PB1-F2	PA		HA					NA		M1	M2
		84	385	627	682	683	47	105	441	65	242	283	321	479	137	296	248	19
JS2018	H7N4	A	**L**	**K**	G	**T**	**S**	**L**	**M**	R	**Q**	**D**	**E**	**M**	**V**	**R**	**I**	**Y**
Ck2018	H7N4	A	I	E	G	**T**	N	F	L	R	H	N	K	I	A	K	M	C
A/Ck/103/2018	H7N4	A	**L**	E	G	K	**S**	**L**	**M**	R	H	**D**	**E**	I	A	**R**	M	C
Dk2018	H7N4	A	I	E	**R**	K	N	F	L	R	H	N	K	I	A	K	M	C
A/Dk/8/2018	H7N4	A	I	E	**R**	K	N	F	L	R	H	N	K	I	A	K	M	C
A/Dk/12/2018	H7N4	**T**	I	E	G	K	N	F	L	R	H	N	K	I	A	K	M	C
A/Dk/13/2018	H7N4	A	I	E	G	K	N	F	L	R	H	N	K	I	A	K	M	C
A/Dk/16/2018	H7N4	A	I	E	**R**	K	N	F	L	**I**	H	N	K	I	A	K	M	C
A/Anhui/1/2013	H7N9	A	I	**K**	G	**T**	**S**	F	**M**	R	H	**D**	**R**	I	**T**	**Q**	**L**	C
A/Shanghai/1/2013	H7N9	A	I	**K**	G	**T**	**S**	F	**M**	R	H	**D**	**R**	I	**T**	**Q**	**L**	C
A/Shanghai/1/2006	H5N1	A	I	E	G	**T**	**S**	F	**M**	K	**F**	**Y**	**N**	**F**	A	H	M	C

JS: Jiangsu; Ck: chicken; Dk: duck. Bold characters: mutated residues.

## Discussion

Our data showed that the HA and NA genomes of a novel human H7N4 isolate were not homologous to those of previously published H7N4 viral sequences, but closer to the HA of an H7N7 and the NA of an H8N4 AIV from two distant and different geographic regions, indicating a probable reassortment (Figs 1 & 3). Our analysis was consistent with a recent report [9], which, however, did not further examine the origin of the internal genomic segments. Historically, emerging avian H5N1 and H7N9 viruses have acquired internal gene segments from endemic H9N2 viruses co-circulating in the region and it would have been no surprise if the current H7N4 viruses followed the same path of acquiring internal gene segments [10, 11]. In this study, however, our data concluded that the internal gene segments were not derived from circulating H9N2 viruses. Our study showed that all six internal gene segments of the novel H7N4 viruses were likely derived from H7N7 or H8N4 viruses, except for PB1, which may be acquired from an H5N1 virus (Fig 2). We hypothesized that the contributing H7N7 virus was local, while the H8N4 donor came from a distant region. Clearly, the six internal segments were not derived from a single or even two avian viruses. We therefore suggested a multi-reassortment model for the genesis of these novel H7N4 virus, although we are unable to show an exact temporal order of the reassortment events.

Scanning the genome of the human and related avian H7N4 viruses, we characterized seventeen mutations potentially involved in human adaptation or enhanced viral replication. First, mutations on HA may cause conformational changes that impact receptor-binding affinity. All the H7N4 and some of the H7N9 viruses studied had an avian-like “QSG” H3 numbering motif. It is likely that this motif was not the only determinant of host specificity [15]. Second, E627K in PB2 of the human H7N4 virus may have been a determinant of host range [15]. The new K683T mutation in PB2 may also be crucial for increased polymerase activity in mammalian cells. The K683T mutant conferred enhanced polymerase activity at low temperatures and promoted efficient replication in the upper respiratory tract [15]. Third, PB1-F2 regulated multiple host responses through various mechanisms as previous reported [16]. We identified an N47S mutation in PB1-F2 that might behave similarly to the N66S mutation which inhibited an early interferon response due to spatial proximity [17]. Last, C19Y mutation in M2, with tyrosine in place of cysteine, could limit disulfide linkage to the neighbor cysteine 17 and prevent the formation of M2 dimerization [18].

A major limitation, at this stage, is the unavailability of this emerging human H7N4 virus isolate, since the patient was treated with oseltamivir which prevented isolation of the virus. It is unknown whether this virus had acquired human-to-human transmissibility. Therefore, further study using reverse genetics approach to rescue this virus and recreate the single-point mutants listed in this study, followed by *in vitro* and “in-ferret” infections to understand virus adaptation, is demanded. Data from such attempts will further elucidate the nature of this novel human H7N4 virus and broaden our understandings about how it adapts and causes a severe infection in the human host while retaining low pathogenicity in avian species.

Although since this case, no new H7N4 strains have emerged and no evidence of the H7N4 human-to-human transmission has occurred, the risk that thisH7N4 virus could re-emerge and cause outbreaks -should not be underestimated, especially given the fact that the H7N4 virus was asymptomatic among avian species, similar to the circulating H7N9 virus. In fact, two months after this human case appeared in China, a new H7N4 virus was detected in aquatic birds in Cambodia, which is on the migratory flyway. However, none of the genomic segments of the Cambodian H7N4 virus were similar to those of JS2018. That the novel human H7N4 virus in China may be more a strain of fitness for human infection remains to be studied [19]. Active surveillance and predictive evolutionary studies of the novel H7N4 viruses should be enhanced in East and Southeast Asia.
